# Investigation of the pharmacological treatment patterns of Chinese patients with major depressive disorder under real-world settings using multi-channel sequence analysis

**DOI:** 10.3389/fpsyt.2023.1089504

**Published:** 2023-04-06

**Authors:** Tao Wu, Sijia Dong, Lu Yang, Hong Qiu, Hong Qiu, David Mellor, Jun Chen, Yifeng Xu

**Affiliations:** ^1^Global Epidemiology, Office of Chief Medical Officer, Johnson & Johnson, Beijing, China; ^2^Global Epidemiology, Office of Chief Medical Officer, Johnson & Johnson, Shanghai, China; ^3^Clinical Research Center and Division of Mood Disorders, Shanghai Mental Health Center, Shanghai Jiao Tong University School of Medicine, Shanghai, China; ^4^Global Epidemiology, Office of Chief Medical Officer, Johnson & Johnson, Titusville, NJ, United States; ^5^School of Psychology, Deakin University, Melbourne, VIC, Australia; ^6^Center for Excellence in Brain Science and Intelligence Technology, Chinese Academy of Sciences, Shanghai, China; ^7^Shanghai Key Laboratory of Psychotic Disorders, Shanghai, China

**Keywords:** major depressive disorder, antidepressants, multi-channel sequence analysis, treatment patterns, electronic health records

## Abstract

**Background:**

Despite many treatment guidelines available now, the treatment patterns of major depressive disorder (MDD) in China haven't been well-understood due to complexity and diversity.

**Aim:**

To describe pharmacological treatment patterns of MDD patients in real-world settings using electronic health records from a major psychiatric hospital in China.

**Methods:**

MDD patients (18–65 years, ICD-10: F32.x, F33.x) newly initiated single antidepressant (AD) in 2015 were enrolled, the date of first AD prescription during the study period was defined as index date, and eligible patients were followed up to 1 year. Treatment patterns were revealed and analyzed using multi-channel sequence analysis (MCSA), considering patients' chronological sequences (in days) of AD prescription, cumulative treatment step(s), and polypharmacy usage during the follow-up.

**Results:**

This study (*n* = 5,003) identified four types of MDD treatment patterns. The first type (1-time treatment) represents the largest proportion of patients (73.6%, *n* = 3,686), followed by the second type (6-month consistent treatment) and third type (long-term, consistent treatment) collectively accounted for 20.6% (*n* = 1,031) of patients, by contrast the last type (long-term, inconsistent treatment) made up the rest 5.7% (*n* = 286) of patients while exhibiting the most complicated treatments patterns. The choice of AD was dominated by selective serotonin reuptake inhibitors (SSRIs), while treatment duration spent in polypharmacy spanned at 2.8%, 16.4%, 2.0%, and 36.5% over the four types, respectively.

**Conclusion:**

Treatment patterns reflecting real-world pharmacological treatment practices of MDD in China were revealed using MCSA. The observed discrepancies between real-world practice and treatment guidelines provided additional insights in improving the clinical management of MDD.

## Introduction

Major depressive disorder (MDD) is a common psychiatric disorder worldwide, characterized by depressed mood, decreased energy, and loss of interest or pleasure in activities once enjoyed. As a leading cause of disability and disease burden globally, MDD accounted for 8.2% of all disability-adjusted life years lost, according to the Global Burden of Disease Study 2010 (1, 2). A 2019 cross-sectional epidemiological study of a nationally representative sample in China found that MDD was the most prevalent mood disorder, with a lifetime prevalence of 3.4% and a 12-month prevalence of 2.1% (3). The diagnosis and treatment of MDD are guided by several international clinical practice criteria, such as those established by the American Psychiatric Association (APA), the Canadian Network for Mood and Anxiety Treatment (CANMAT), and the British National Institute for Health and Care Excellence (NICE) (4–6). In China, the first clinical guidelines for MDD were published in 2003 by the Chinese Society of Psychiatry (CSP), which were updated to conform to international practice and drug development in 2015 (7). Recommended in these guidelines, pharmacological treatment is the major approach for managing MDD, particularly for moderate to severe cases, and antidepressants (ADs) are the recommended medications in clinical practice globally and in China. However, despite this recommendation, extensive evidence has shown that the effectiveness of ADs is generally comparable (4, 5, 8). Consequently, most guidelines provide general recommendations for pharmacological treatment of MDD patients, which leads to variation in clinical practice. The selection of ADs is based on factors such as clinical features of MDD, safety, and tolerability of alternative medications (4). Moreover, a study of MDD treatment pathways, which summarized all the medication sequences from a large, diverse population, found that not only were the pathways highly divergent, but 11% of the sampled population had completely unique pathways (9). Such complexity in pathways was also illustrated in another study across four US claims databases as half of the patients receiving initial AD were prescribed a variety of different medications in subsequent treatments, and no single AD class dominated (10). The diversity in the treatment pathways poses a challenge to understanding the actual treatment pathways in clinical practice and in optimizing treatment recommendations. To the best of our knowledge, previous studies on MDD treatment pathways were mostly limited by their complicated nature with little attention paid to patterns beyond the initial switch.

Developed from the social sciences, sequence analysis (SA) is a method used to study the sequences of event status in longitudinal data, such as career and family development in life trajectories. Notably, this method quantifies dissimilarities between trajectories using edit distance (a metric derived from counting the common attributes) (11). This method has since been adopted in health research (12) for novel applications to the healthcare pathways of various diseases (13–16). Gauthier et al. (17) extended the concept of SA from single trajectories to multi-channel (trajectories) sequence analysis (MCSA) to enable analysis of multiple trajectories simultaneously, thus improving its applicability in more complex situations. MCSA, therefore, provides a new approach for analyzing treatment patterns. In this study, we aim to expand the current knowledge of MDD treatment pathways among Chinese patients by utilizing the novel approach of MCSA based on Electronic Health Record (EHR) collected from the Shanghai Mental Health Center (SMHC), Shanghai Jiao Tong University, a WHO Collaborating Center in China. We hypothesized that the AD prescriptions, the cumulative treatment changes, and the polypharmacy usage, representing three channels of chronological trajectories, would cover the primary aspects of MDD pathways while balancing computational complexity and interpretability of results. Our study offers a unique perspective that could enhance the current understanding of the MDD treatment pathways.

## Methods

### Data source

This study utilized EHR collected at the SMHC, a first-class tier III public hospital that specializes in providing mental health services to patients with routine care needs, as well as those with severe and complex mental disorders from all over China. Relevant clinical information, including patient demographics (e.g., gender and date of birth), diagnoses [coded using the International Classification of Diseases, 10th Revision, Clinical Modification (ICD-10-CM)], drug prescription, and quantity as well as the dates of medical services were collected. AD treatments were captured using drug prescription information. This retrospective database study was approved by the Institutional Review Boards, Shanghai Mental Health Center (2019-18R). All information was de-identified and extracted from the hospital information system (HIS) into a validated and standardized EHR database by the hospital information technology department thus written informed consent from patients was waived.

### Study setting

This is a descriptive study of the 1-year treatment pathways among Chinese MDD patients who newly initiated single AD treatment in 2015. The ADs eligible for initial treatment are listed in Supplementary Table 1 (excluding antipsychotics). We included 1 year of data prior to and after the treatment initiation to ensure complete baseline and follow-up observations. A patient's first observed prescription in 2015 with no record of AD use in the previous year was defined as the index prescription, and the date was defined as the index date.

Patients were eligible for inclusion in the study if they met the following criteria:

(a) male or female,
(b) aged between 18 and 65 at the index date,
(c) had been diagnosed with major depressive disorder (ICD-10 code: F32.x, F33.x) in baseline,
(d) prescribed with at least one AD in 2015.

Patients were excluded if they:

(a) received a diagnosis of bipolar disorder (ICD-10 code: F30.x, F31.x) or schizophrenia (ICD-10 code: F20.x) during the baseline and follow-up period,
(b) had been administered electroconvulsive therapy (ECT) in baseline,
(c) had been diagnosed with organic disease(s) of the central nervous system (ICD-10 code: F00.x- F09.x) in baseline and follow-up,
(d) patients with multiple AD medications (two or more) on the index date.

The focus of this study was on patients who were prescribed a single AD on their index date since monotherapy is universally recommended as the initial regimen. Patients were followed up to 365 days from the index date or the end of the first episode, whichever came first. An episode was considered ended when there had been 120 days with no diagnosis of depression and no dispensing of AD medication had been observed.

### Multi-channel sequence analysis

We applied MCSA to identify patient groups who received similar treatments across multiple trajectories. We selected and obtained the trajectories (channels), converted each trajectory into sequence data for the study period, and performed hierarchical clustering to identify patterns based on all trajectories.

In this study, three trajectories were chosen for channel-sequence building: AD class, treatment step, and polypharmacy usage. Based on an individual's prescriptions during follow-up, ADs were grouped into pre-specified AD classes (Supplementary Table 1), selective serotonin reuptake inhibitors (SSRIs), serotonin and norepinephrine reuptake inhibitors (SNRIs), tricyclic antidepressants (TCAs), noradrenergic and specific serotonergic antidepressants (NaSSAs), other antidepressants (other ADs), and antipsychotics (AP). The treatment step described cumulative treatment changes since the index date. Specifically, a change (step) was identified if there was a (1) Switch: The patient AD medication(s) was changed by initiating one or more different AD(s) and discontinuing the prior AD(s) within a 30-day period, the new AD(s) must have a minimum supply of 30 days, and (2) Add-on: The patient's treatment regimen was modified by augmenting it with one or more other AD(s) for a minimum of 30 days. The AD in the initial prescription was defined as a step 1 regimen. If a later switch or add-on was observed, then the patient entered the step 2 regimen. The treatment step was ordered as step 1, step 2, and step 3 or greater. Polypharmacy status was grouped into single-drug or multi-drug. Of note, the AD class, treatment step, and polypharmacy usage were all determined at the drug level. Each channel described above was converted into chronological sequence data at daily level starting from the index date to the end of follow-up. For those whose first episode terminated earlier than 1 year, their treatment status beyond the termination was considered as “treatment discontinuation,” resulting in all individual sequences spanning 365 days for subsequent analysis. Prescription gaps were imputed by the last observable value of the respective channels. In the end, three parallel sequences of length 365 days for each individual were obtained. The pairwise 3-channel dissimilarities between individuals were computed using the Longest Common Subsequence (LCS) distance, which takes position-shift into account. In other words, we considered ordered sequences such as “SSRIs-SNRIs-SSRIs” and “SNRIs-SSRIs-SNRIs” as close since they were similar when position-shift was allowed even though they differ entirely position-wise. Ward-linkage hierarchical clustering was applied to the dissimilarity matrix to identify homogeneous groups of sequences in joint consideration of the three channels. We used the average silhouette width (ASW) to assess the clustering's homogeneity, and a value of 0.51 or above generally indicates reasonable partition (18). The final partitions were chosen based on clustering homogeneity, size of the clusters, and interpretability (Supplementary material) (19).

The resulting clusters were characterized by demographics, clinical characteristics, and drug utilization. Overall utilization of ADs during follow-up was analyzed by drug class. We identified treatment changes, including subsequences and transitions in each cluster. Transitions refer to immediate changes between any adjacent sequences (e.g., “SSRI>SNRI”), while subsequences allow for additional changes in between (e.g., “SSRI-SNRI” with zero or more intervening treatments). Transition focus on immediate changes, while subsequences enable identification of more generalized treatment patterns (20).

Finally, given the evidence suggesting that there may be variations in symptoms and prevalence of MDD between patients of different sex (21), a subgroup analysis by gender was performed to further explore whether such variation could lead to different treatment pattern for males and females. Summary statistics for categorical variables (frequency and percentage), continuous variables [mean, standard deviation (sd)] were presented. Statistical analyses were performed using SAS software, version 9.4 (SAS Institute, Cary, North Carolina). Multi-channel sequence analysis was performed with the TraMineR package (Version 1.8-8) (22), multi-channel distribution was visualized with seqHMM package (23), and clustering quality was evaluated with the WeightedCluster package (18) using R version 4.0.0 (24).

## Results

### Demographics and clinical characteristics

From the SMHC database, a total of 10,371 MDD patients who newly initiated MDD treatment during 2015 were identified (Supplementary Figure 1). Of these, 312 were excluded due to a diagnosis of schizophrenia, bipolar disorder, or organic disease of the CNS; 12 were excluded due to having been administered ECT before the first identified prescription of AD; 1,785 patients did not meet the pre-specified age criterion; 3,259 patients were excluded due to being prescribed multiple ADs on the index date. This left 5,003 patients who met all criteria and were included in the study cohort. Among the eligible patients, the mean age was 39.1 years (sd, 13.11), 65.6% were female and most were in outpatient care on their index date (99.3%; Table 1). The mean follow-up duration was 100.4 days (sd, 120.9), during which 4,451 of the patients (89.0%) terminated their treatments within 1 year.

**Table 1:** Demographic and clinical characteristics.

	**Overall (*****N*** = **5,003)**		**Cluster 1 (*****N*** = **3,686)**		**Cluster 2 (*****N*** = **474)**		**Cluster 3 (*****N*** = **557)**		**Cluster 4 (*****N*** = **286)**	
**Age mean (sd)**	39.1	13.11	38.4	12.85	40.9	13.23	40.4	13.96	42.1	13.72
**Age group** ***n*** **(%)**										
18–30	1,597	31.9	1,223	33.2	135	28.5	169	30.3	70	24.5
31–40	1,397	27.9	1,068	29.0	122	25.7	137	24.6	70	24.5
41–50	778	15.6	559	15.2	87	18.4	79	14.2	53	18.5
51–65	1,231	24.6	836	22.7	130	27.4	172	30.9	93	32.5
**Female** ***n*** **(%)**	3,283	65.6	2,426	65.8	300	63.3	385	69.1	172	60.1
**Place of service on index date** ***n*** **(%)**									
Outpatient	4,970	99.3	3,668	99.5	470	99.2	555	99.6	277	96.9
Inpatient	33	0.7	18	0.5	4	0.8	2	0.4	9	3.1

### Identification of the treatment patterns

**Figure 1:**
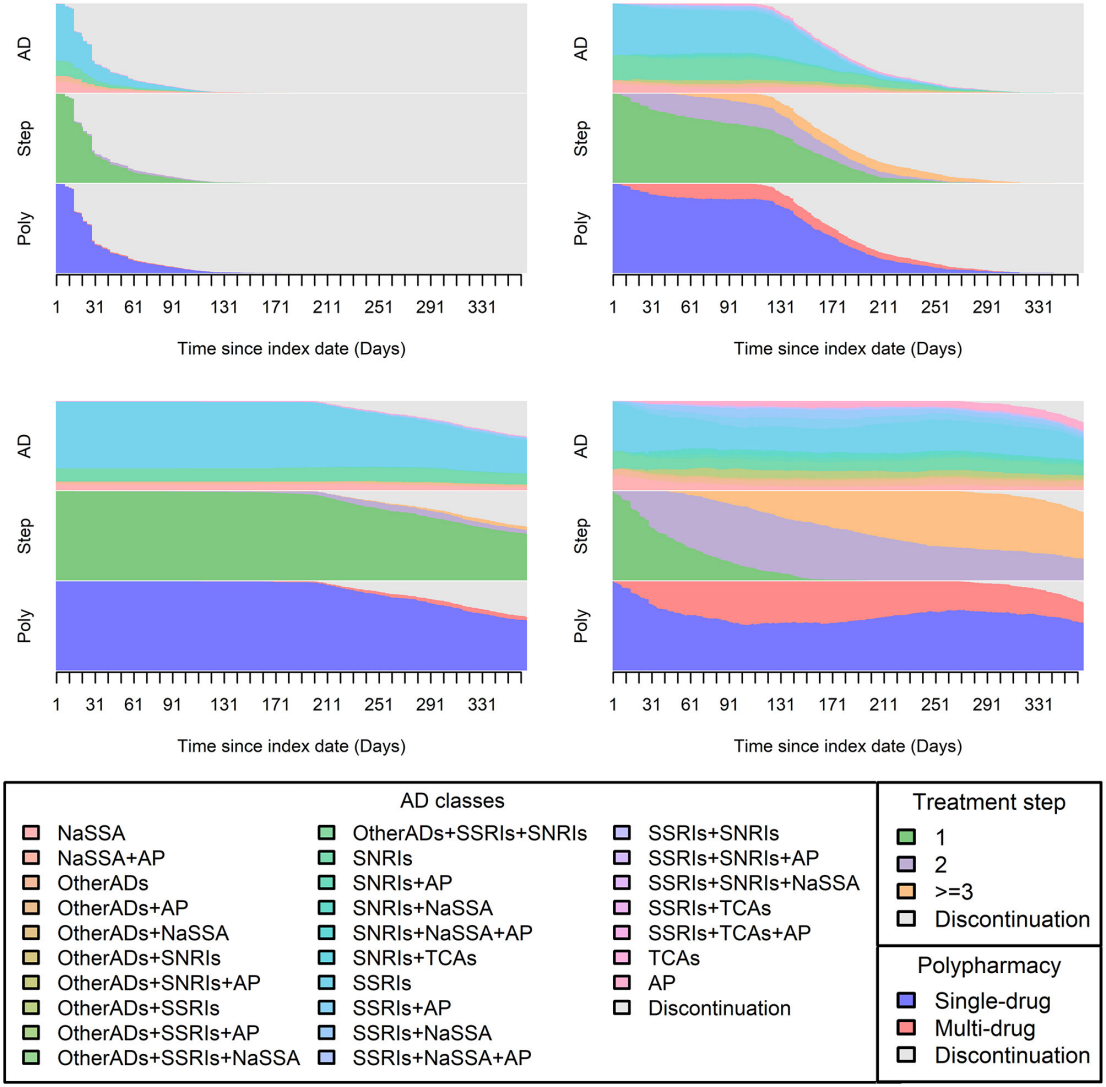
Distribution plot of each cluster. In this figure, visualization of 1-year treatment pathways from all patients by four identified clusters was plotted and each consisted of information from the three pre-defined channels [AD class, treatment step (Step), and polypharmacy usage (Poly)]. The X-axis displays chronological order (365 days), and the daily sequence values obtained from each channel were plotted according to their corresponding proportions, which were displayed on the Y-axis.

Among the 5,003 subjects, 1,282, 1,017, and 791 distinct sequences were identified in the AD class channel, treatment step channel, and polypharmacy usage channel, respectively (the channel overview and top 10 patterns of each channel are presented visually in Supplementary Figures 2, 3). Ward hierarchical clustering (process detailed in Supplementary Figures 4, 5) yielded four homogeneous groups, which provided the optimal results in terms of sequence separation quality, size, and interpretability. Figure 1 displays the treatment pathway of each cluster by three channels. The ASW value of 0.65 suggests that the partitioning was reasonable.

### Demographics and clinical characteristics by clusters

The demographics and clinical characteristics of each cluster are summarized in Table 1. Cluster 1 patients are slightly younger than those in other clusters while patients in Cluster 4 are slightly older. Females were dominant in all clusters; with cluster 3 having the highest proportion (69.1%) and cluster 4 the lowest (60.1%). With the exception of cluster 4 (96.9%), more than 99% of patients in the first three clusters were outpatients on the index date.

### Treatment pattern characteristics of each cluster

Overall, the four clusters represented four different patterns. Cluster 1, the largest yet most uniform cluster, which accounted for over 70% of the patients (*N* = 3,686), can be characterized by a common early discontinuation of the treatment with a mean time to stop of 35.0 days. SSRIs were the dominant AD class during the follow-up (20.6 days spent on SSRIs on average). Furthermore, patients mainly used single-drug (on average for 34.0 days, 97%) and remained in the first treatment step (no switches or add-on therapies; 32.5 days), with a mean total treatment step count of 1.1. Compared to cluster 1, cluster 2 (*N* = 474, 10%) had a longer mean time to discontinuation of 180.8 days. In terms of treatment, SSRIs and SNRIs were the most dominant AD classes; with patients spending 80.7 and 46.3 days on them, respectively. In addition, cluster 2 demonstrated a similar pattern in terms of treatment step and polypharmacy usage, with a majority of time spent on single-drug therapy (151.1 days, 84%), and an average of 120.2 days spent in the first treatment step. The mean treatment steps for this cluster was 1.7. Cluster 3 (*N* = 557, 12%) differed significantly from the previous two clusters in several ways. Firstly, the treatment duration was by far the longest (averaging 333.0 days). Secondly, it had the highest percentage of patients on SSRIs and the fewest on SNRIs and NaSSAs. For example, 74.9% of the patients in cluster 3 initiated AD therapy on an SSRI and spent an average of 236.8 days on it (cluster 1 and cluster 2 spent 20.6 and 80.7 days on an SSRI, respectively). Cluster 3 also had the highest percentage of time spent in both the first treatment step (317.9 days, 95%) and single-drug therapy (326.2 days, 98%) during the treatment period, with a mean treatment step of 1.2. Cluster 4 (*N* = 286, 5.7%) was the smallest cluster, yet its pattern was notably different from previous clusters. Firstly, this cluster was characterized by its longest mean time to stop of 357.3 days. Treatment involved a wide variety of AD classes, with SSRIs being the most commonly prescribed class, with an average of 118.4 days spent, and 85% of follow-up time (302.9 days) was spent in step 2 treatment and above. Furthermore, multi-drug use was more prevalent in this cluster than in the other three clusters (mean time of 130.4 days spent on multi-drug therapy, 36%). Correspondingly, the mean treatment step count of 3.3 for patients in this cluster was greater than the other clusters (Table 2).

**Table 2:** Drug utilization and healthcare utilization by clusters.

	**Cluster 1 (*****N*** = **3,686)**		**Cluster 2 (*****N*** = **474)**		**Cluster 3 (*****N*** = **557)**		**Cluster 4 (*****N*** = **286)**	
**Follow-up duration (days) mean (sd)**	35.0	27.62	180.8	46.25	333.0	49.64	357.3	18.66
**Patient with treatment discontinuation** ***n*** **(%)**	3,686	100.0	474	100.0	223	40.0	68	23.8
**AD class** ^*^								
**Initiating AD class** ***n*** **(%)**								
SSRI	2,353	63.8	274	57.8	417	74.9	161	56.3
SNRI	623	16.9	133	28.1	82	14.7	57	19.9
NaSSA	455	12.3	49	10.3	38	6.8	46	16.1
**Stopping AD class** ***n*** **(%)**								
SSRI	2,298	62.3	223	47.0	171	76.7	31	45.6
SNRI	601	16.3	112	23.6	16	7.2	10	14.7
NaSSA	444	12.0	24	5.1	18	8.1	/	<1.0%
**AD class ever used** ***n*** **(%)**								
SSRI	2,374	64.4	296	62.4	422	75.7	207	72.4
SNRI	637	17.3	161	34.0	93	16.7	95	33.2
NaSSA	472	12.8	56	11.8	42	7.5	64	22.4
**Time (days) spent during follow-up mean (sd)**								
SSRI	20.6	24.69	80.7	75.18	236.8	145.96	118.4	121.17
SNRI	5.5	15.23	46.3	76.28	53.3	124.09	44.9	86.15
NaSSA	4.4	14.99	12.5	42.30	22.7	83.21	21.4	57.25^**^
**Treatment step**								
**Cumulative number of steps mean (sd)**	1.1	0.23	1.7	1.00	1.2	0.57	3.3	1.27
**Stopping treatment step** ***n*** **(%)**								
Step 1	3,513	95.3	272	57.4	194	87.0	0	0.0
Step 2	165	4.5	90	19.0	18	8.1	16	23.5
Step ≥3	8	0.2	112	23.6	11	4.9	52	76.5
**Time (days) spent during follow-up mean (sd)**								
Step 1	32.5	25.03	120.2	68.21	317.9	63.66	54.4	42.81
Step 2	2.3	11.29	38.7	55.57	11.5	35.15	166.4	160.21
Step ≥3	0.1	1.85	21.9	46.83	3.5	16.59	136.5	108.42
**Polypharmacy usage**								
**Stopping polypharmacy usage** ***n*** **(%)**								
Single-drug	3,612	98.0	398	84.0	212	95.1	49	72.1
Multiple-drug	74	2.0	76	16.0	11	4.9	19	27.9
**Time (days) spent during follow-up mean (sd)**								
Single-drug	34.0	26.88	151.1	56.01	326.2	53.31	226.9	112.18
Multiple-drug	1.0	6.84	29.7	55.58	6.8	24.79	130.4	113.53

^*^Top 3 classes covering the majority of AD classes were listed.

^**^NaSSA ranked the forth class in cluster 4, and the third class was antipsychotics (mean: 23.1 days, sd: 64.79 days).

### Stopping treatment, typical patterns of each cluster

All patients in cluster 1 and cluster 2 discontinued treatments during the follow-up, whereas only 223 (40.0%) and 68 (23.8%) of the patients in cluster 3 and cluster 4 discontinued the treatment within 1 year, respectively. For those who discontinued treatment, SSRIs, SNRIs, and NaSSA were the most used before treatment ended, and single-drug usage was significantly more common than multi-drug use (Table 2).

Despite the differences in treatment durations regarding the treatment steps, most patients in cluster 1, 2, and 3 remained in step 1 during treatment. However, clusters 2 and 4 were characterized by increase in the number of treatment steps, with 42.6 and 100% of patients advancing to step 2 and higher proportion of patients progressing to step 3 than in clusters 1 and 3 (Table 2). The most common changes in therapy (i.e., subsequences and transitions) identified from each cluster are listed in Supplementary Table 2. Notably in cluster 4, the most frequent switching pattern during follow-up was from SSRIs to combinations of SSRIs with other drugs or ADs, observed in ~12% of the patients, and most switches were sequential. Moreover, 10.1% of the patients switched from SSRIs to other antipsychotics later amidst the follow-up.

Regarding polypharmacy usage during follow-up, patients in clusters 1 and 3 predominantly used single drugs, while in cluster 2, 29.5% of patients switched from single-drug to multiple-drug therapy, and 15.2% of the patients switched from single-drug to multiple-drugs and then returned to a single-drug. A comparable pattern was observed in cluster 4 (Supplementary Table 2).

### Treatment pattern and characteristics by gender

Subgroup analysis was conducted by gender, revealing four clusters for both female (cluster 1–4: 2,192, 567, 380, and 144 patients) and male (cluster 1–4: 1,120, 335, 180, and 85 patients). The first cluster remained the largest for both genders, accounting for over 65% of patients, with an average time to discontinuation of 27.6 days for females and 29.0 days for males. Cluster 2 and 3 demonstrated similar patterns to the overall population, with ~½-year treatment in cluster 2 and ~1-year treatment in cluster 3 for both genders. Cluster 4 also exhibited similar patterns, with more advanced treatment steps and more polypharmacy usage. No major differences were observed between genders. Basic characteristics were also consistent with the overall population, with patients in cluster 4 tending to be slightly older and have lower proportion of outpatient visits on the index date. Detailed information on cluster distribution and patient characteristics by cluster for both genders can be found in Supplementary Figures 6, 7 and Supplementary Table 3.

## Discussion

The present study described the 1-year MDD pharmacological treatment pathway among patients who newly initiate treatment from one of the largest psychiatric hospitals in China. As one of the most recognized mental health centers in China, the clinical practice observed from SMHC has reasonable representativeness of MDD treatment. Despite the complexity of the potential treatment patterns from real-world practice as well as the difficulty in utilizing more information in the data analysis, we used sequence-based approach to lessen the gap between limited analysis methods and the understanding of MDD treatment patterns.

Among the 5,003 MDD patients included from the SMHC database, the majority were female with an average age of 39.1 years. The cohort was treated with ADs for an average duration of 100 days. One of the attractive features of using EHR database is the ability to provide information reflecting clinical practice. To extend beyond simple description, we identified four types of MDD treatment patterns based on the observed real-world clinical pathways. These patterns revealed important insights into how MDD patients are treated in China. However, it should be noted that the interpretation of such patterns requires caution as they are a mix of clinical practice and real-world complexities. Our aim was to describe and summarize the clinical characteristics and implications of the MDD treatment in China based on these patterns.

The first type, comprising more than 70% of patients, discontinued treatment after a mean duration of 35 days, indicating a pattern of 1-time treatment since 1-month interval between visits is usually scheduled for MDD patients in the SMHC practice. This apparent rapid cessation of treatment is at odds with the guidelines that recommend that treatment with the same AD continue 4–9 months in a continuous treatment phase after remission (4). Indeed, other studies in South Korea (25) and Taiwan, China (26) have reported a mean duration of pharmaceutical treatment for depression of 152 days and 1.57 years, respectively. In the present case, except for a possible rapid response in mildly symptomatic patients, there may be other causes for the observed very early discontinuation. There is no medical referral mechanism in China, and the public tends to go directly to top-level hospitals because of the high-quality medical resources (27, 28). Thus, tertiary hospitals may provide consultancy for patients from other provinces who may return to their local hospitals for maintenance treatments. This may explain the sparse visit pattern observed in top hospitals, such as seen in SMHC. In addition, the short treatment observed in majority of Chinese MDD patients may be caused by the low public awareness of mental health diseases such as depression as well as the negative attitude toward seeking professional psychological help (29). A recent review identified several key barriers of poor mental health help-seeking behavior among the Chinese population, including self-reliance, low perceived need, lack of affordability, etc. (30). A recent national survey revealed a concerning fact that only 9.5% of participants with a 12-month history of MDD sought mental health services, and only 0.5% were considered adequately treated with ADs or mood stabilizers in specialized mental health service (31). On the other hand, the second and third types of patterns consisted of 20% of patients who shared similar treatment patterns as the first type, except for a relatively longer continuation of treatment for about 6 months and 1 year, respectively. This observed maintenance may reflect typical patterns of regular and effective MDD treatment. The last type of pattern was characterized by a prolonged treatment duration, during which mixed usage of ADs, more frequent prescription changes, as well as multi-drug usage were observed, this may indicate the ineffectiveness of treatment or severe symptoms. While this pattern included <6% of all patients, this pattern may be most relevant to treatment resistance and may encompass a larger percentage of patients in other settings.

The prescription of AD classes in our study showed similarities from previous findings, with SSRIs being the most commonly prescribed class. This is consistent with previous studies in the US (32), Europe (33), East Asia (34), and China (35, 36). In the present study, more than 60% of patients in the SMHC sample initiated SSRI treatment, which is in line with the first-line treatment recommended in most guidelines, including Chinese guideline for MDD treatment updated in 2015 (6, 7). Nevertheless, nearly one-third of the patients were excluded from the study due to having multi-AD prescriptions on the index date; it is possible these patients had already started treatment elsewhere, or they had more severe symptoms or were refractory to first treatments and were referred to this tertiary care center. In terms of the last regimen before treatment discontinuation, SSRIs also remained the dominant AD in all clusters, indicating their effectiveness in treatment to some extent, if remission were manifested as the last treatment prior to cessation of therapy (at least in a database). The observed dominance of SSRIs from treatment initiation to cessation is in line with the well-established efficacy, safety, and tolerability. However, this also highlights the limited flexibility in drug choice in clinical practice is revealed (37). A more individualized approach to drug selection, taking into account clinical features, medication characteristics, and patient preferences may be beneficial in practice. Further study is required to investigate the clinical implications of these findings, especially for the last type of treatment pattern where treatment-resistance was noted. Moreover, the subgroup analysis by gender revealed that the treatment pattern identified in males and females were similar to those observed in the overall population, and no significant differences were observed between genders.

To the best of our knowledge, this is the first study to comprehensively identify MDD treatment patterns in China. The Chinese guidelines for treating MDD provide general recommendations rather than a treatment algorithm that outlines specific and sequenced treatment strategies for psychiatrists. As a result, MDD patients in Chinese clinical practice follow diverse treatment pathways. Therefore, a comprehensive understanding of the patients' actual treatment is necessary, and pattern identification is crucial for clinical guidance. Such an approach can offer insight into developing appropriate treatment algorithms or strategies appropriate tailored to Chinese patients. From the patterns identified in our study, we found about 17% of patients continue long term treatment for 1 year (maybe longer since the follow-up period was truncated at 1 year in this study) and two-third of them exhibited complex patterns with frequent changes in antidepressant treatment, which complied to the common perception of treatment-resistant depression. Also, the observed large proportion of 1-time treated patient implies the potential need of effective strategies to promote positive attitude toward treatment awareness. This helps manage healthcare utilization resources and better recognize the potential disease burden of treatment-resistant depression in practice.

In light of the above, applying sequence analysis to treatment data has great potential to provide insights into the MDD treatment pathways visually and analytically. Two features often complicate understanding complex treatment pathways from the real-world setting: the high diversity, which often makes traditional summary statistics inapplicable, and the difficulty of utilizing the pathway data more efficiently. Researchers are often forced to focus on cross-sectional analysis with low-dimensional information to reduce diversity and complexity. Compared to conventional analysis, a sequential analysis approach may lead to a more thorough characterization of treatment pathways by looking at all treatments beyond the index drug, analysis concerning time, treatment switches, drug utilization, and common status changes on both overall and individual levels. Such characteristics could be coupled with selected outcomes and further utilized to provide additional clinical insights. Our results confirmed that such strategies could generate meaningful results.

### Limitations

This study has several limitations. First, the majority of the patients had short treatment patterns, as observed in cluster 1. This may limit the present study's ability to capture long-term treatment. However, in addition to the identified potential reasons for the observed short treatment, this limitation may be also informative in reflecting the current real-world practice of MDD treatment in China, highlighting the gap between the actual practice and guidelines. On the other hand, the SMHC database used in this study lacks confirmed treatment discontinuation information, which is a general issue when conducting EHR studies in China and elsewhere. Nonetheless, our definition of treatment discontinuation is sufficient to provide an initial and broad understanding of true treatment terminations. To further address this issue, one may attempt to identify and exclude the high mobility of patients between hospitals. Secondly, the data source from a single hospital may have limited our estimation of exposure and follow-up as it would have captured patients whose MDD history was complex, difficult to treat, but ultimately were followed longer term by another clinician. In China, Class 1 level III hospital offer services to the general population from all over the country, providing up-to-date medical care for patients, including routine health care and treatment for more severe and complex cases. SMHC is one of the leading class 1 level III mental health hospitals in China, the EHR could provide valuable insight into the real-world treatment patterns of MDD with reasonable representativeness in terms of patients' characteristics and treatment patterns. Future research is warranted to further verify the results from our study. Thirdly, prescribed medication cannot equate to the actual treatment of patients, while the assumption that medication prescribed is as consumed as directed is a common approach in EHR-based studies. Finally, in MCSA the choice of channels as well as the quantification methods used for assessing similarities could affect the subsequent clustering results and lead to different interpretations. These items should be chosen based on analysis interests and study design. Although several quality assessment criteria are widely available, the choice of the number of clusters is subjective and arbitrary, and no single best solution is indicated. Instead, the process is balanced by cluster separation quality, size, and interpretability, therefore caution should be exercised when examining the clustering results. Overall, while these limitations should be considered when interpreting the study's results, we believe that this study's findings contribute valuable insight into the real-world treatment patterns of MDD in China.

## Conclusion

In conclusion, this study provided new insights into the real-world treatment practices and disease management among MDD patients in China with a novel application of multi-channel sequence analysis. Further work should be done to validate our findings in different data sources.

## Data availability statement

The original contributions presented in the study are included in the article/Supplementary material, further inquiries can be directed to the corresponding authors.

## Author contributions

TW conceived the original idea and study design, supervised on methodology and technical details, performed data curation, and drafted original manuscript. SD contributed to the study design, methodology, implementation of the research, and drafted original manuscript. LY performed analysis and produced the figures. HQ (4th Author) (SMHC) extracted and validated the data. HQ (5th Author) supervised the project and contributed to conceptualization and methodology. DM aided in interpreting the results and edited the manuscript. JC and YX provided critical consultation from clinical perspective and contributed to manuscript writing. All authors contributed to editing and commenting on the final manuscript. All authors contributed to the article and approved the submitted version.

## References

[1] Ferrari AJ Charlson FJ Norman RE Flaxman AD Patten SB Vos T. The epidemiological modelling of major depressive disorder: Application for the Global Burden of Disease Study 2010. PLoS ONE. (2013) 8:e69637. 10.1371/journal.pone.0069637 23922765 PMC3726670

[2] Murray CJL Vos T Lozano R Naghavi M Flaxman AD Michaud C. Disability-adjusted life years (DALYs) for 291 diseases and injuries in 21 regions, 1990–2010: A systematic analysis for the Global Burden of Disease Study 2010. Lancet. (2012) 380:2197–223. 10.1016/S0140-6736(12)61689-4 23245608

[3] Huang Y Wang Y Wang H Liu Z Yu X Yan J. Prevalence of mental disorders in China: A cross-sectional epidemiological study. Lancet Psychiatry. (2019) 6:211–24. 10.1016/S2215-0366(18)30511-X 30792114

[4] Kennedy SH Lam RW McIntyre RS Tourjman SV Bhat V Blier P. Canadian Network for Mood and Anxiety Treatments (CANMAT) 2016 clinical guidelines for the management of adults with major depressive disorder: Section 3. Pharmacological treatments. Can J Psychiatry. (2016) 61:540–60. 10.1177/0706743716659417 27486148 PMC4994790

[5] American Psychiatric Association Steering Committee on Practice Guidelines,. Practice Guideline for the Treatment of Patients With Major Depressive Disorder. 3rd ed. (2010). Available online at: https://psychiatryonline.org/pb/assets/raw/sitewide/practice_guidelines/guidelines/mdd.pdf (accessed 4 November 2022).

[6] NICE. Depression in Adults: Recognition and Management. (2009). Available online at: https://www.nice.org.uk/guidance/cg90/chapter/1-Guidance (accessed 4 November 2022).

[7] Feng Y Xiao L Wang W-W Ungvari GS Ng CH Wang G. Guidelines for the diagnosis and treatment of depressive disorders in China: The second edition. J Affect Disord. (2019) 253:352–6. 10.1016/j.jad.2019.04.104 31078835

[8] Gartlehner G Hansen RA Morgan LC Thaler K Lux LJ Van Noord M. AHRQ Comparative Effectiveness Reviews. In Second-Generation Antidepressants in the Pharmacologic Treatment of Adult Depression: An Update of the 2007 Comparative Effectiveness Review. Rockville, MD: Agency for Healthcare Research and Quality (US). (2011). 22299185

[9] Hripcsak G Ryan PB Duke JD Shah NH Park RW Huser V. Characterizing treatment pathways at scale using the OHDSI network. Proc Natl Acad Sci USA. (2016) 113:7329–36. 10.1073/pnas.1510502113 27274072 PMC4941483

[10] Kern DM Cepeda MS Defalco F Etropolski M. Treatment patterns and sequences of pharmacotherapy for patients diagnosed with depression in the United States: 2014 through 2019. BMC Psychiatry. (2020) 20:4. 10.1186/s12888-019-2418-7 31900133 PMC6942399

[11] Navarro G. A guided tour to approximate string matching. ACM Comput Surveys. (2001) 33:31–88. 10.1145/375360.375365

[12] Brzinsky-Fay C Ritschard G Studer M Sequence analysis and related approaches. innovative methods and applications: Springer 2018. Eur J Popul. (2019) 35:429–31. 10.1007/s10680-018-09513-w

[13] Jay N Nuemi G Gadreau M Quantin C. A data mining approach for grouping and analyzing trajectories of care using claim data: The example of breast cancer. BMC Med Inform Decis Mak. (2013) 13:130. 10.1186/1472-6947-13-130 24289668 PMC4220620

[14] Le Meur N Gao F Bayat S. Mining care trajectories using health administrative information systems: The use of state sequence analysis to assess disparities in prenatal care consumption. BMC Health Serv Res. (2015) 15:200. 10.1186/s12913-015-0857-5 25976089 PMC4436876

[15] Moreno-Black G Boles S Johnson-Shelton D Evers C. Exploring categorical body mass index trajectories in elementary school children. J Sch Health. (2016) 86:495–506. 10.1111/josh.12402 27246674 PMC5055398

[16] Roux J Grimaud O Leray E. Multichannel sequence analysis: An innovative method to study patterns of care pathways. Application to multiple sclerosis based on French Health Insurance data. Revue d'Épidémiologie et de Santé Publique. (2018) 66:S430–1. 10.1016/j.respe.2018.05.534

[17] Gauthier J-A Widmer ED Bucher P Notredame C. Multichannel sequence analysis applied to social science data. Sociol Methodol. (2010) 40:1–38. 10.1111/j.1467-9531.2010.01227.x

[18] Studer M. WeightedCluster Library Manual: A Practical Guide to Creating Typologies of Trajectories in the Social Sciences With R. LIVES Working Papers. (2013). p. 24. 10.12682/lives.2296-1658.2013.24

[19] Emery K Berchtold A. Comparison of two approaches in multichannel sequence analysis using the Swiss Household Panel. Longit Life Course Stud. (2022) 2022:1–32. 10.1332/175795921X16698302233894 37874200

[20] Gabadinho A Ritschard G Studer M Müller N. Mining Sequence Data in R With the TraMineR Package: A User's Guide. Laboratory of Demography, University of Geneva. (2011). Available online at: http://traminer.unige.ch

[21] Vetter JS Spiller TR Cathomas F Robinaugh D Brühl A Boeker H. Sex differences in depressive symptoms and their networks in a treatment-seeking population—a cross-sectional study. J Affect Disord. (2021) 278:357–64. 10.1016/j.jad.2020.08.074 33002727 PMC8086368

[22] Gabadinho A Ritschard G Müller N Studer M. Analyzing and visualizing state sequences in R with TraMineR. J Stat Softw. (2011) 40:1–37. 10.18637/jss.v040.i04

[23] Jounim H Satu H. seqHMM: Mixture hidden Markov Models for Social Sequence Data Other Multivariate, Multichannel Categorical Time Series. R Package Version 1.0.14. (2019). Available online at: https://cran.r-project.org/package=seqHMM (accessed 4 November 2022).

[24] R Core Team. R: A Language Environment for Statistical Computing. Vienna: R Foundation for Statistical Computing. (2020). Available online at: https://www.R-project.org/ (accessed 4 November 2022).

[25] Kim N Cho SJ Kim H Kim SH Lee HJ Park CHK. Epidemiology of pharmaceutically treated depression and treatment resistant depression in South Korea. PLoS ONE. (2019) 14:e0221552. 10.1371/journal.pone.0221552 31442296 PMC6707549

[26] Fife D Feng Y Wang MY Chang CJ Liu CY Juang HT. Epidemiology of pharmaceutically treated depression and treatment resistant depression in Taiwan. Psychiatry Res. (2017) 252:277–83. 10.1016/j.psychres.2017.03.006 28288438

[27] Bao Y Fan G Zou D Wang T Xue D. Patient experience with outpatient encounters at public hospitals in Shanghai: Examining different aspects of physician services and implications of overcrowding. PLoS ONE. (2017) 12:e0171684. 10.1371/journal.pone.0171684 28207783 PMC5312958

[28] Shi J Chi C Gong X Chen C Yu W Huang J. Examining health disparities and characteristics in general practice utilization: Based on outpatient data from 2014 - 2018 in Shanghai. BMC Fam Pract. (2020) 21:74. 10.1186/s12875-020-01146-5 32349689 PMC7190008

[29] Chen P Liu XJ Wang XQ Yang BX Ruan J Liu Z. Attitude toward seeking professional psychological help among community-dwelling population in China. Front Psychiatry. (2020) 11:417. 10.3389/fpsyt.2020.00417 32477190 PMC7240032

[30] Shi W Shen Z Wang S Hall BJ. Barriers to professional mental health help-seeking among Chinese adults: A systematic review. Front Psychiatry. (2020) 11:442. 10.3389/fpsyt.2020.00442 32508688 PMC7251144

[31] Lu J Xu X Huang Y Li T Ma C Xu G. Prevalence of depressive disorders and treatment in China: A cross-sectional epidemiological study. Lancet Psychiatry. (2021) 8:981–90. 10.1016/S2215-0366(21)00251-0 34559991

[32] Olfson M Marcus SC. National patterns in antidepressant medication treatment. Arch Gen Psychiatry. (2009) 66:848–56. 10.1001/archgenpsychiatry.2009.81 19652124

[33] Bauer M Monz BU Montejo AL Quail D Dantchev N Demyttenaere K. Prescribing patterns of antidepressants in Europe: Results from the Factors Influencing Depression Endpoints Research (FINDER) study. Eur Psychiatry. (2008) 23:66–73. 10.1016/j.eurpsy.2007.11.001 18164600

[34] Uchida N Chong MY Tan CH Nagai H Tanaka M Lee MS. International study on antidepressant prescription pattern at 20 teaching hospitals and major psychiatric institutions in East Asia: Analysis of 1898 cases from China, Japan, Korea, Singapore and Taiwan. Psychiatry Clin Neurosci. (2007) 61:522–8. 10.1111/j.1440-1819.2007.01702.x 17875031

[35] Yu Z Zhang J Zheng Y Yu L. Trends in antidepressant use and expenditure in six major cities in China From 2013 to 2018. Front Psychiatry. (2020) 11:551. 10.3389/fpsyt.2020.00551 32765307 PMC7378967

[36] Zhang Y Becker T Kösters M. Preliminary study of patterns of medication use for depression treatment in China. Asia Pac Psychiatry. (2013) 5:231–6. 10.1111/appy.12022 23857675

[37] Lochmann D Richardson T. Selective serotonin reuptake inhibitors. In:M Macaluso SH Preskorn, editors, Antidepressants: From Biogenic Amines to New Mechanisms of Action. Cham: Springer International Publishing. (2019). p. 135–44. 10.1007/164_2018_172 30838457

