# Supplementary material for: Investigation of the pharmacological treatment patterns of Chinese patients with major depressive disorder under real-world settings using multi-channel sequence analysis
Source: Front Psychiatry. 2023 Apr 6;14:1089504. doi: 10.3389/fpsyt.2023.1089504 (PMC10115953; doi:10.3389/fpsyt.2023.1089504)
Supplement: Supplementary file 1 [file Data_Sheet_1.docx]

**Supplementary materials**

**Table S1. Antidepressants medications and therapeutic class included in the study**

| Drug class | Antidepressants |
| --- | --- |
| Selective serotonin reuptake inhibitors (SSRIs) | Escitalopram, Citalopram, Fluoxetine, Fluvoxamine, Paroxetine, Sertraline |
| Serotonin and noradrenalin reuptake inhibitors (SNRIs) | Duloxetine, Milnacipran, Venlafaxine |
| Noradrenergic and specific serotonergic antidepressants (NaSSA) | Mirtazapine |
| Tricyclic antidepressants (TCAs) | Amitriptyline, Clomipramine, Doxepin, Imipramine, Tianeptine |
| Other antidepressants (other ADs) | Bupropion, Reboxetine, Trazodone, Maprotiline, Mianserine，Agomelatine |
| Antipsychotics (AP) | Aripiprazole, Quetiapine, Risperidone, Lithium, Olanzapine, Methylphenidate, Ziprasidone |

**Choice of cluster partition**

The process of clustering and the final partitions were chosen based on clustering homogeneity, size, and interpretability. Homogeneity shows the quality of clustering which reflect the similarity of the pattern of the patients identified as one group. Homogeneity could be measured by the average silhouette width (ASW) and usually a value of 0.51 or above generally indicates reasonable partition. Size is the patient number included in one cluster, and too few patients in a cluster (an extreme case is that partition could be proceed until each patient form a cluster) would be difficult to summarize the pattern and lose clinical significance. Interpretability is the clinical meaning of treatment pattern considered in terms of drug used, change frequency and polypharmacy usage (consistent with the channels). No single best solution is indicated (mentioned in the last point of limitation) but balance the three aspects to get reasonable partitions with meaningful pattern characteristics.

**Figure S1. patient selection flowchart**


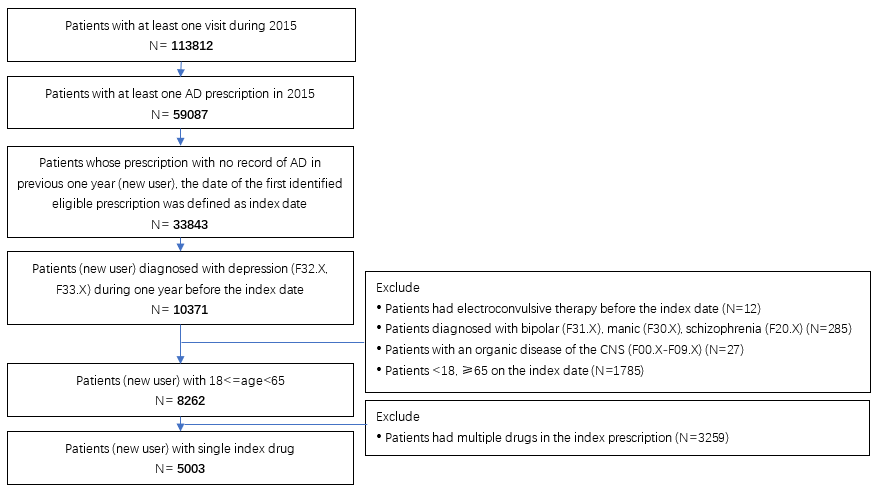


**Figure S2.** Overall treatment pathways by 3 channels. (AD class, treatment step, polypharmacy usage)


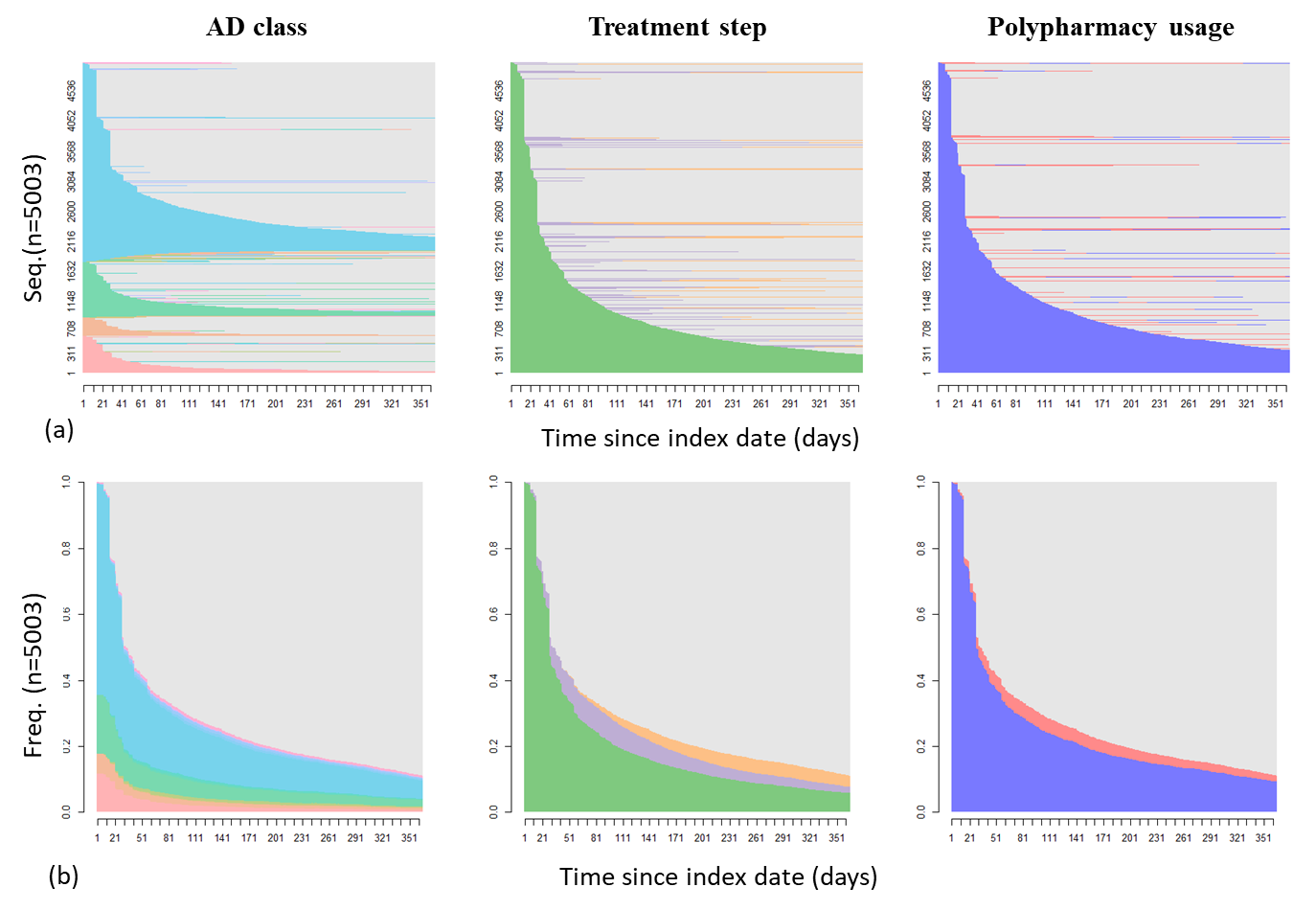


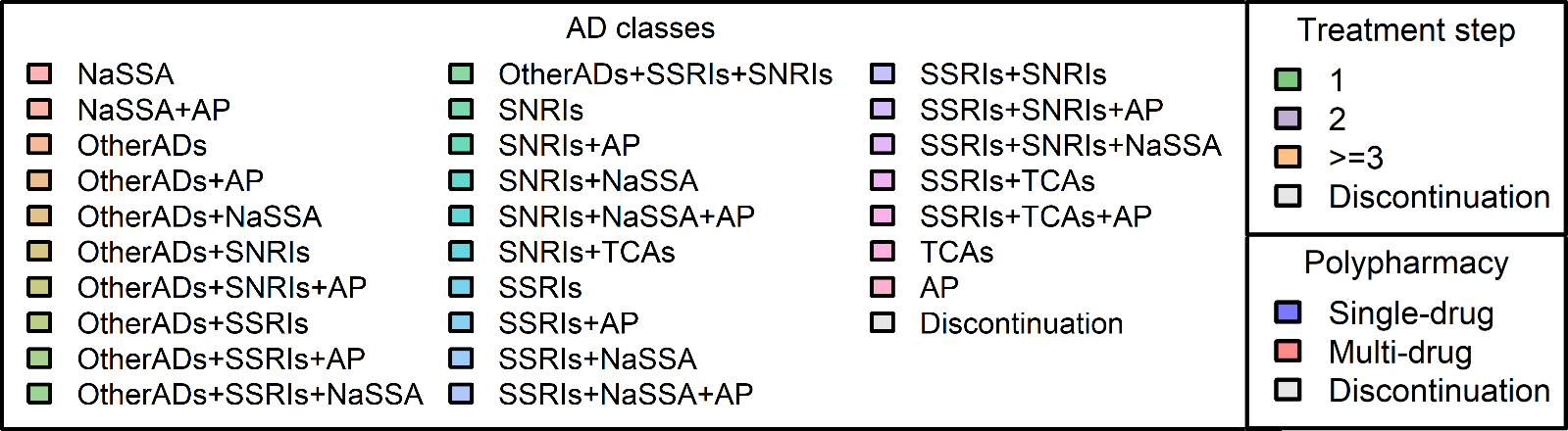


****Figure legend applies to all subsequent plots.***

**Figure S2(a). Individual sequence plots** Reading from left right, each horizontal line represents a complete course of treatment sequence up to 365 days. The corresponding states on any specific days are represented by different colors assigned in Figure 1 of main text. From the sequence plots, most patients showed similar pattern in terms of the 3 channels with various treatment length. On the other hand, quite a few exceptions that deviate from the major patterns can be observed at various treatment length.

**Figure S2(b).** **Distribution plots** Based on Figure S2a, individual-level information was aggregated with respect to each channel and plotted in Figure S2b, in which the daily values obtained from each channel were plotted according to their corresponding proportions. Similarly, reading from left to right, the proportion of different states and its change in three channels during 1-year post index are displayed.

**Figure S3**. Top patterns by 3 channels


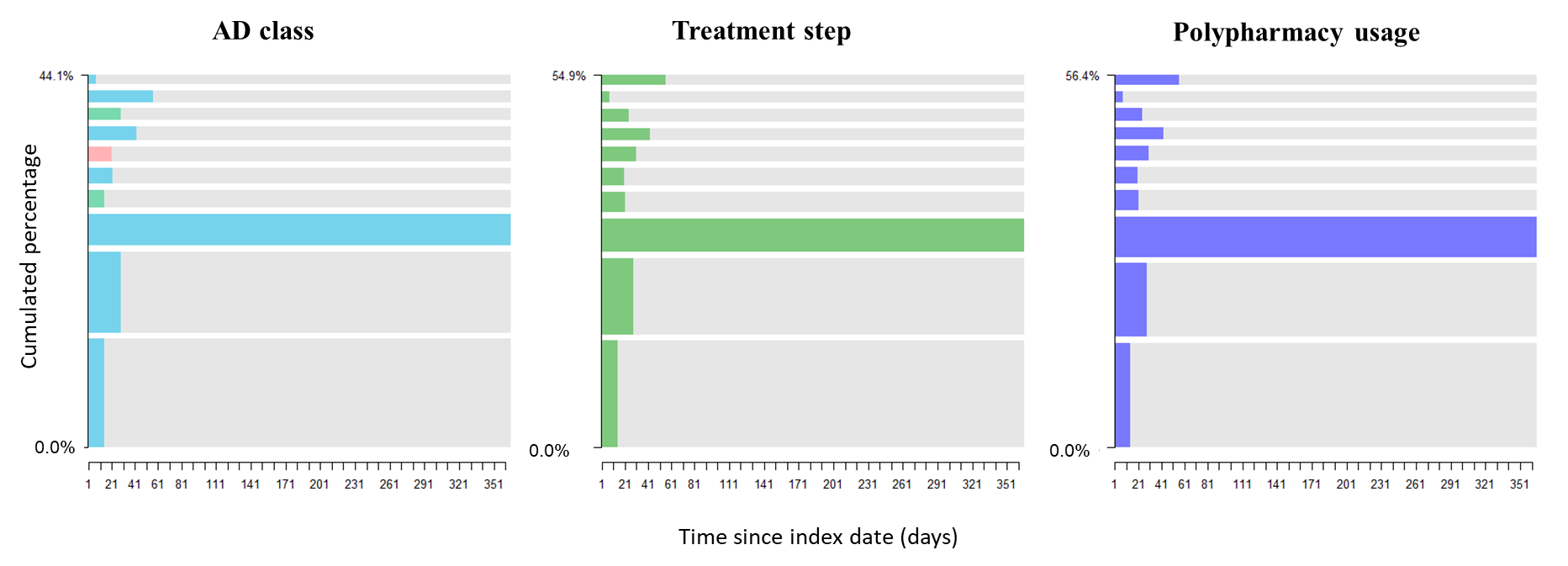


The 10 most frequent sequences overall were plotted in Figure S3. In AD class channel, the top 10 sequences made up of 44.1% of the patients, in which most initiated with SSRIs. In the first and second most common sequences, patients stopped medication very early (on day 14 and day 28) and accounted for 15.1% and 11.3% of all sequences in the channel respectively. The third most common sequence shows 220 patients (4.4%) who initiated with SSRIs and continued treatment with SSRI till the end of follow up. Similarly, in treatment step channel, the top 10 sequences summarized 54.9% of the patients and no treatment change was observed regardless of treatment stop time. Of note, 291 patients (5.8%) in the third most sequence stayed in the first treatment step until the end of study period. Furthermore, in polypharmacy usage status channel the top 10 sequences covered 56.4% of the patients and similar pattern was observed where single-drug usage was the dominant pattern, 362 patients (7.2%) in the third most frequent sequence used single-drug throughout the 365 days.

**Figure S4.** Hierarchical clustering procedure (distribution plot)


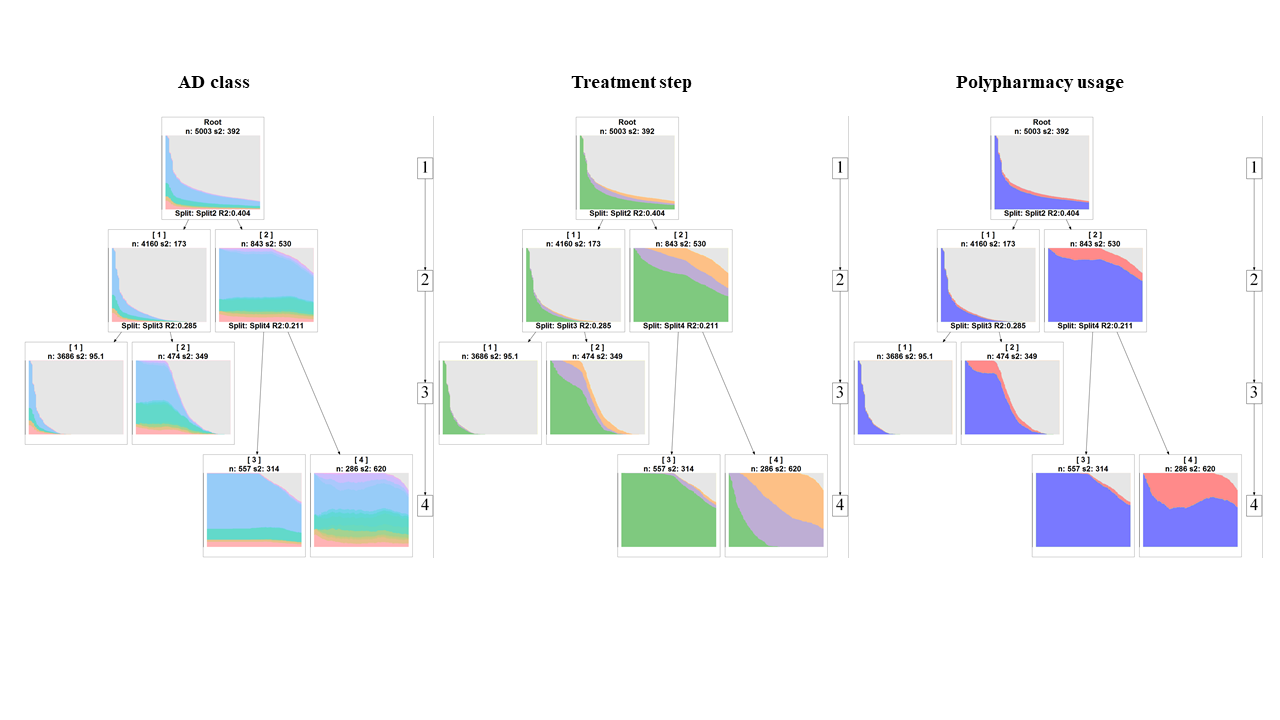


The hierarchical clustering process of each channel resulting the final 4 clusters are illustrated by distribution plot at each step in Figure S4.

**Figure S5a.** Hierarchical clustering procedure (individual sequence plot)


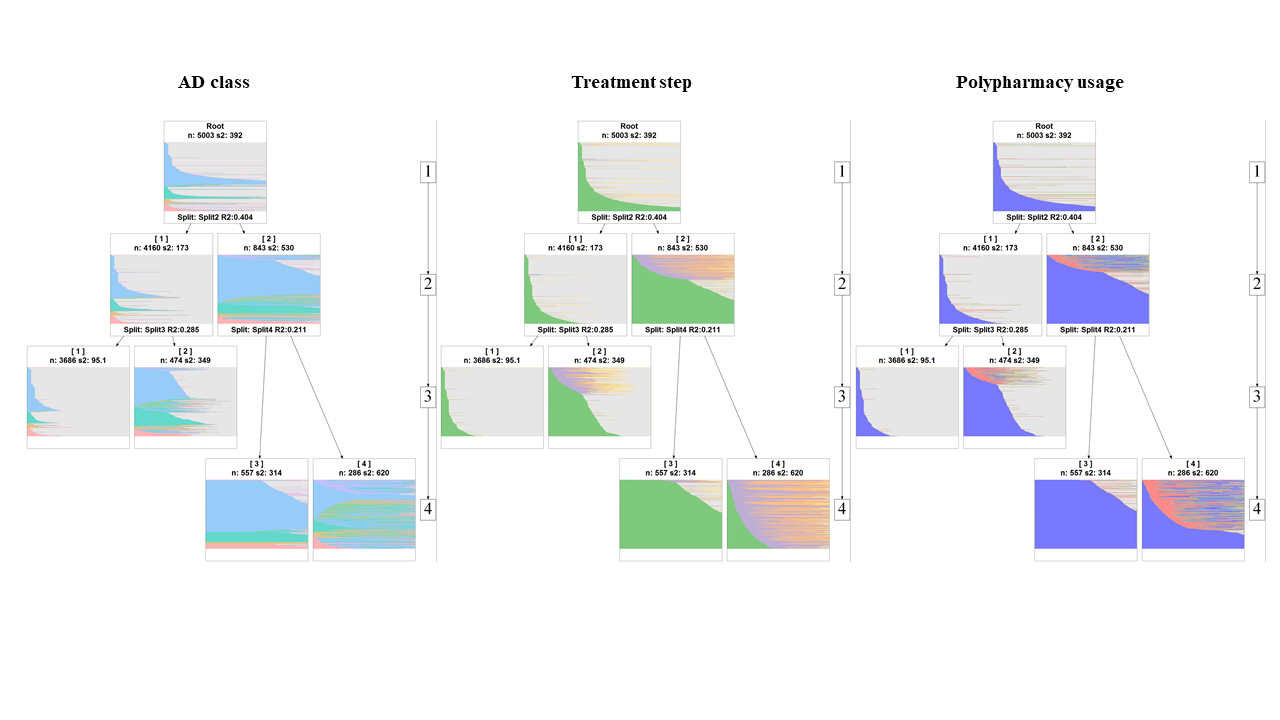


The hierarchical clustering process of each channel resulting the final 4 clusters are illustrated by individual sequence plot at each step in Figure S5a.

**Figure S5b.** Individual sequence plot of each cluster


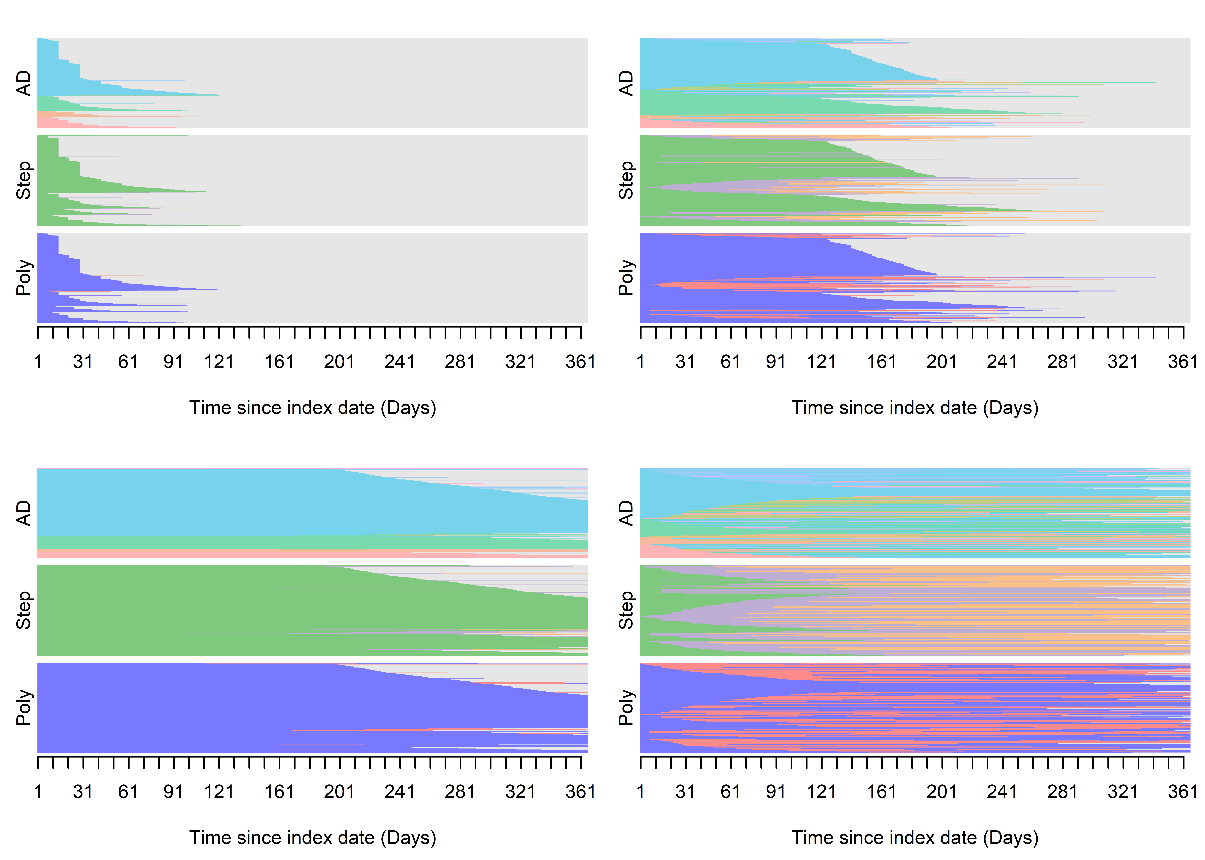


**Table S2.** Selected frequent subsequences and transitions of each clusters.

|  | Cluster 1 (N=3686) | Cluster 2 (N=474) | Cluster 3 (N=557) | Cluster 4 (N=286) |
| --- | --- | --- | --- | --- |
| **Top 3 subsequences n, (%)** |  |  |  |  |
| SSRI-(SSRI+others) | <1% | 12, (2.6) | 14, (2.5) | 39, (13.6) |
| SSRI-(SSRI+other ADs) | <1% | 22, (4.6) | <1% | 37, (12.9) |
| SSRI-others | <1% | 13, (2.7) | 7, (1.3) | 29, (10.1) |
|  |  |  |  |  |
| **Top 3 transitions n, (%)** |  |  |  |  |
| SSRI>(SSRI+others) | <1% | 11, (2.3) | 12, (2.2) | 38, (13.3) |
| SSRI>(SSRI+other ADs) | <1% | 21, (4.4) | <1% | 36, (12.6) |
| SSRI>others | <1% | 11, (2.3) | <1% | 9, (3.1) |
|  |  |  |  |  |
| **Top transitions of polypharmacy usage status n, (%)** |  |  |  |  |
| Single-drug switch to multi-drug | 78, (2.1) | 140, (29.5) | 48, (8.6) | 213, (74.5) |
| Start with single-drug, switch to multi-drug, then single-drug | <1% | 72, (15.2) | 16, (2.9) | 164, (57.3) |
| Start with single-drug, switch to multi-drug, end with single-drug | <1% | 64, (13.5) | 6, (1.1) | 35, (12.2) |
| Start with single-drug, switch to multi-drug, then single-drug, then multi-drug | <1% | <1% | <1% | 58, (20.3) |
| Start with single-drug, switch to multi-drug, then single-drug, then multi-drug, then single-drug | <1% | <1% | <1% | 26, (9.1) |

We attempted to further profile each cluster by summarizing the top transitions observed as well as top subsequences within each cluster. In our attempt, transition was defined specifically as the change of states between any adjacent sequences, for example the transition “SSRI>SNRI” refers to the immediate switch from SSRI to SNRI without additional states in-between. On the other hand, subsequence was specifically referred as a flexible version of transition where additional states are allowed in-between two transitions. In this setting, “SSRI-SNRI” would mean any sequences that begin with SSRI and end with SNRI while zero or more states in-between. Hence, transition focuses on immediate changes between any two states whereas subsequence enables the identification of more generalized treatment pattern.

**Subgroup analysis by gender**

**Treatment pattern by gender**

**Figure S6a. Distribution plot of each cluster (female) Figure S6b. Individual sequence plot of each cluster (female)**


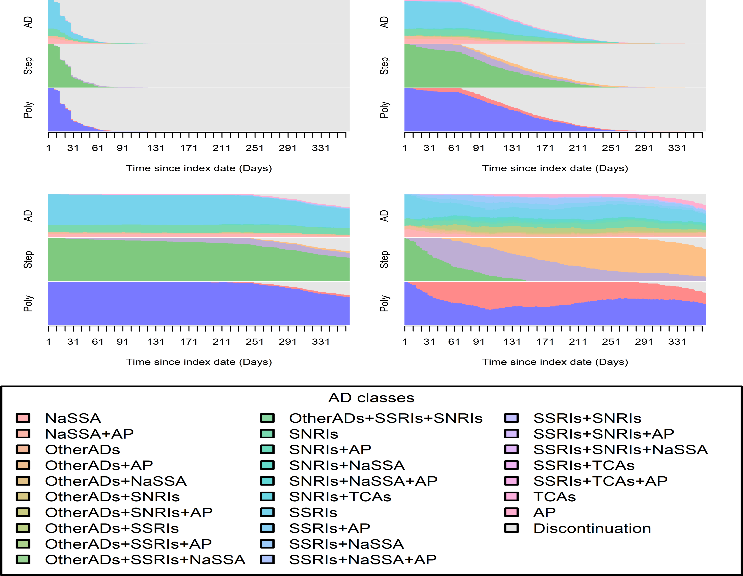

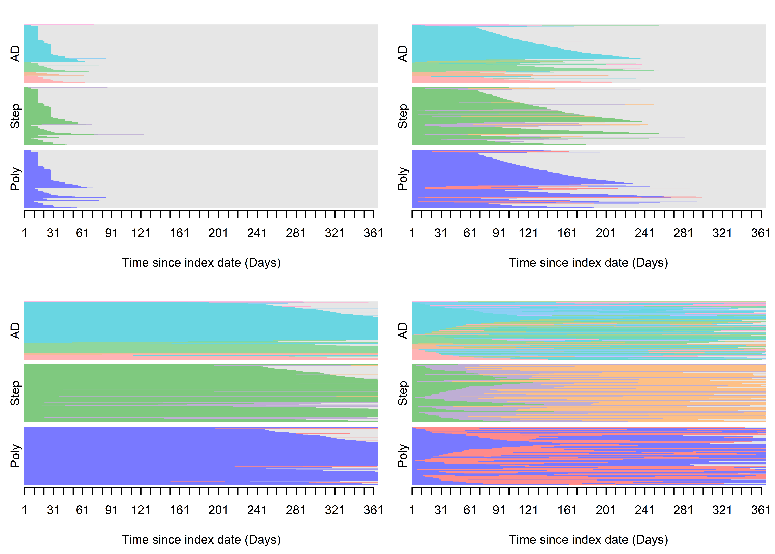


**Figure S7a. Distribution plot of each cluster (male) Figure S7b. Individual sequence plot of each cluster (male)**


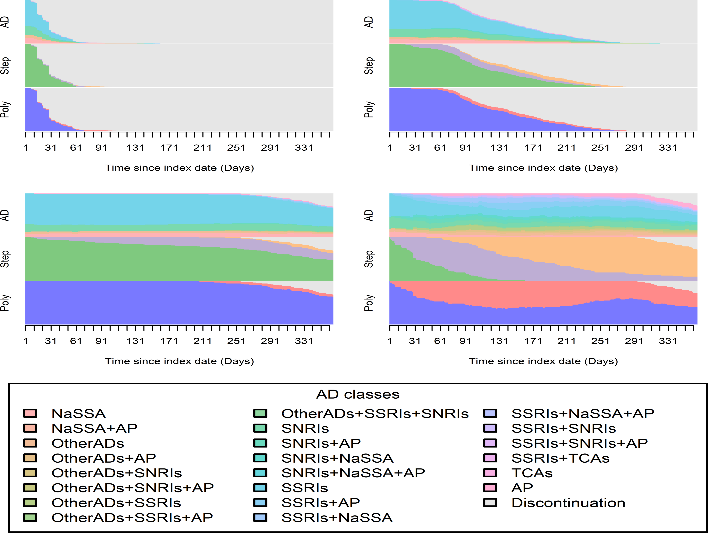

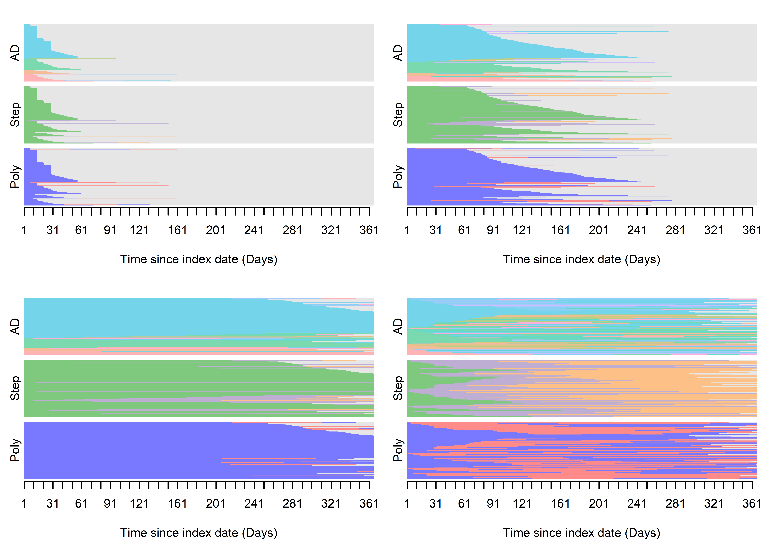


**Table S3. Basic characteristics by gender and by cluster**

| **Female (N=3283)** | | | | | | | | |
| --- | --- | --- | --- | --- | --- | --- | --- | --- |
|  | **Cluster 1 (N=2192)** | | **Cluster 2 (N=567)** | | **Cluster 3 (N=380)** | | **Cluster 4 (N=144)** | |
| **Age mean, (sd)** | 38.9 | 12.89 | 41.5 | 13.49 | 41.4 | 14 | 42.4 | 13.89 |
| **Age group n, (%)** |  | |  | |  | |  | |
| 18-30 | 697 | 31.8 | 146 | 25.7 | 103 | 27.1 | 37 | 25.7 |
| 31-40 | 635 | 29.0 | 142 | 25.0 | 91 | 23.9 | 29 | 20.1 |
| 41-50 | 320 | 14.6 | 103 | 18.2 | 60 | 15.8 | 30 | 20.8 |
| 51-65 | 540 | 24.6 | 176 | 31.0 | 126 | 33.2 | 48 | 33.3 |
| **Place of service on index date n, (%)** |  | |  | |  | |  | |
| Outpatient | 2179 | 99.4 | 562 | 99.1 | 378 | 99.5 | 141 | 97.9 |
| Inpatient | 13 | 0.6 | 5 | 0.9 | 2 | 0.5 | 3 | 2.1 |
| **Follow-up duration (days) mean, (sd)** | 27.6 | 17.16 | 148.2 | 54.74 | 346.2 | 33.84 | 355.9 | 20.47 |
| **Cumulative number of steps mean, (sd)** | 1.0 | 0.20 | 1.4 | 0.79 | 1.3 | 0.60 | 3.6 | 1.20 |
| **Male (N=1720)** | | | | | | | | |
|  | **Cluster 1 (N=1120)** | | **Cluster 2 (N=335)** | | **Cluster 3 (N=180)** | | **Cluster 4 (N=85)** | |
| **Age mean, (sd)** | 36.8 | 12.34 | 37.7 | 13.16 | 40.5 | 13.65 | 41.4 | 13.36 |
| **Age group n, (%)** |  | |  | |  | |  | |
| 18-30 | 413 | 36.9 | 128 | 38.2 | 52 | 28.9 | 21 | 24.7 |
| 31-40 | 339 | 30.3 | 87 | 26.0 | 51 | 28.3 | 23 | 27.1 |
| 41-50 | 170 | 15.2 | 57 | 17.0 | 24 | 13.3 | 14 | 16.5 |
| 51-65 | 198 | 17.7 | 63 | 18.8 | 53 | 29.4 | 27 | 31.8 |
| **Place of service on index date n, (%)** |  | |  | |  | |  | |
| Outpatient | 1117 | 99.7 | 334 | 99.7 | 179 | 99.4 | 80 | 94.1 |
| Inpatient | 3 | 0.3 | 1 | 0.3 | 1 | 0.6 | 5 | 5.9 |
| **Follow-up duration (days) mean, (sd)** | 29.0 | 20.11 | 146.9 | 59.44 | 350.3 | 27.99 | 355.6 | 18.86 |
| **Cumulative number of steps mean, (sd)** | 1.1 | 0.28 | 1.4 | 0.75 | 1.4 | 0.74 | 3.8 | 1.24 |
